# New-Onset Diabetes Mellitus, Hypertension, Dyslipidaemia as Sequelae of COVID-19 Infection—Systematic Review

**DOI:** 10.3390/ijerph192013280

**Published:** 2022-10-14

**Authors:** Marysia Wrona, Damian Skrypnik

**Affiliations:** 1 Poznan University of Medical Sciences, 61-701 Poznan, Poland; 2 Department of Treatment of Obesity, Metabolic Disorders and Clinical Dietetics, Poznan University of Medical Sciences, 60-569 Poznan, Poland

**Keywords:** COVID-19 sequelae, new-onset diabetes mellitus, new-onset dyslipidaemia, new-onset hypertension, post-COVID, long-COVID

## Abstract

As the population recovers from the coronavirus disease 2019 (COVID-19) pandemic, a subset of individuals is emerging as post-coronavirus disease (post-COVID) patients who experience multifactorial long-term symptoms several weeks after the initial recovery from severe acute respiratory syndrome coronavirus 2 (SARS-CoV-2) infection. The aim of this systematic review is to present the latest scientific reports that evaluate changes in glucose levels, blood pressure readings and lipid profiles after recovery from COVID-19 to verify the hypothesis that new-onset diabetes mellitus, arterial hypertension and dyslipidaemia are a possible sequela of a COVID-19 infection. The open access databases PubMed and Google Scholar were searched. Articles investigating patients with residual clinical signs and biochemical alteration indicating diabetes, hypertension and dyslipidaemia at least a month after recovering from COVID-19 were included. It has been shown that a select number of patients were diagnosed with new-onset diabetes, arterial hypertension and dyslipidaemia after COVID-19 infection. Alterations in glucose levels, blood pressure and lipid profiles months after initial infection shows the importance of considering diabetes mellitus, arterial hypertension and dyslipidaemia as part of the multifactorial diagnostic criteria post-COVID to better provide evidence-based clinical care.

## 1. Introduction

Despite most individuals infected with severe acute respiratory syndrome coronavirus 2 (SARS-CoV-2) recovering within weeks of infection, a subset of individuals experiences post-coronavirus disease (post-COVID) conditions. Post-COVID conditions, also known as long-coronavirus disease (long-COVID), is a multisystem disease which causes patients to experience a cluster of debilitating symptoms four or more weeks after being infected with SARS-CoV-2 [1]. The incidence of post-COVID sequelae is estimated between 10 and 35% [2,3]. The most common reported symptoms of post-COVID are fatigue, headache, insomnia, trouble concentrating, muscle and joint pain and cough; however, the multifaceted, long-term clinical effect of coronavirus disease 2019 (COVID-19) sequelae remains largely unclear [1,4,5].

Emerging evidence shows that new-onset diabetes mellitus, hypertension and dyslipidaemia are detected during the acute phase of a COVID-19 infection, but limited reviews have been conducted concerning similar outcomes occurring concurrently with post-COVID [6,7,8,9]. A detailed analysis of the risk and burden of diabetes, hypertension and dyslipidaemia are needed to develop integrated post-COVID clinical care management. Therefore, this review aims to present the latest scientific reports that evaluate persistent changes in glucose levels, blood pressure readings and lipid profiles several weeks after infection with COVID-19 to verify the hypothesis that new-onset diabetes mellitus, arterial hypertension and dyslipidaemia might be a sequela of COVID-19 infection.

## 2. Materials and Methods

### 2.1. Eligibility Criteria

Studies included in this review were searched for using PubMed (MEDLINE) and Google Scholar. Predefined search terms included multiple combinations of the following: (post-acute sequelae of SARS-CoV-2 OR post-COVID condition OR post-COVID-19) AND (metabolic syndrome OR metabolic comorbidities OR diabetes OR hyperglycaemia OR insulin resistance OR dyslipidaemia OR arterial hypertension) AND (new-onset).

Included articles were original English language articles published from 1 December 2019 to 30 January 2022 investigating patients with residual clinical signs and biochemical alterations at least a month after recovering from COVID-19; specifically, articles investigated fasting glucose and C-peptide levels, blood pressure and lipid profiles. The eligible articles focused on patients over eighteen years of age. Confirmed infections were defined as positive real-time reverse-transcriptase polymerase chain reaction (RT-PCR) results from a nasopharyngeal and/or throat swab. Only studies reaching the statistical significance have been incorporated.

Excluded articles were published before 1 December 2019, non-English language, non-original studies. Articles were ineligible if sample population did not meet long-COVID definition of new or persistent symptoms four weeks after initial infection.

### 2.2. Selection Process

The screening was mechanically performed by checking the publication date, record of patients testing positive for COVID-19 from a nasopharyngeal swab using the RT-PCR test during acute phase, persistent clinical alteration at least a month after recovery and biochemical investigations comparing baseline status to follow-up. The databases searched a total of 1027 records. After reading the abstracts, 916 studies were removed. The browsing led to the elimination of 96 papers, leaving 10 [9,10,11,12,13,14,15,16,17,18]; the selection of records was performed according to eligibility criteria. To analyse the bias, “The ROBIS” device was used (http://www.bristol.ac.uk/media-library/sites/social-community-medicine/robis/ROBIS%201.2%20Clean.pdf (accessed on 30 January 2022)). Characteristics of the studies that were mainly assessed in bias analysis: specifications of the studies met the eligibility criteria, relevance of studies in context of review aim. See Supplementary Figure S1, which presents a PRISMA flowchart of the review process.

### 2.3. Data Collection Process

This review was conducted following Preferred Reporting Items for Systematic Reviews and Meta-analyses (PRISMA) guidelines. Evaluation of the quality of the included studies was based on selecting only published peer-reviewed studies, excluding preprint manuscripts and case reports.

### 2.4. Outcome Measures

The outcome measure was the alteration from baseline and prevalence of diabetes, arterial hypertension, or dyslipidaemia several months after infection with COVID-19.

## 3. Results

### 3.1. New-Onset Diabetes Mellitus after COVID-19 Recovery

Despite apparent clinical recovery at discharge from initial infection with COVID-19, many patients have residual medical problems and persistent hyperglycaemia when evaluated after several months [9]. Table 1 summarises studies investigating persistent abnormal glucose and insulin levels after COVID-19 infection. Results from a continuous glucose monitoring test demonstrated a greater duration of glycaemia above 140 mg/dL, higher mean postprandial glycaemia at 120 min, higher mean blood glucose and higher nadir blood glucose in post-COVID patients than in the healthy control group [9]. Significantly elevated levels of fasting C-peptide, a by-product when insulin is produced and decreased fasting glucose levels six months after COVID-19 recovery proved that COVID-19 can increase the risk of insulin resistance where a patient produces insulin but does not respond in the right way [10]. A longitudinal study of 354 laboratory-confirmed COVID-19 cases showed that ten individuals with no prior comorbidities were newly diagnosed with diabetes mellitus and experienced associated persisting neurological symptoms of burning and pricking sensations at the post-discharge three-month follow-up [11]. Similarly, a large retrospective cohort study showed that over a mean follow-up of 140 days, rates of diabetes (*p* = 0.001) were significantly raised in patients who recovered from COVID-19, with 127 diagnoses per 1000 patient years [12]. Likewise, data from a cohort study of 1733 COVID-19 individuals discharged from a hospital in China showed that fifty-eight patients without a self-reported history of pre-existing diabetes were newly diagnosed with the condition at their six-month follow-up appointment after recovery [13].

### 3.2. New-Onset Arterial Hypertension after COVID-19 Recovery

Table 2 summarises studies investigating the effects of COVID-19 on blood pressure (BP) by re-evaluating patients BP in the medical office during follow-up evaluation months after recovery. The cohort study by Akpek et al., showed that patients’ systolic blood pressure (SBP) and diastolic blood pressure (DBP) were significantly higher one month after COVID-19 infection compared with when admitted to hospital for COVID-19 [14]. Similarly, another cohort study showed that forty (21.6%) patients had uncontrolled blood pressure requiring treatment after twenty-three days post-discharge from the hospital or emergency department (ED) for COVID-19 infection [15]. A case–control study from Egypt that compared clinical signs and biochemical changes in 120 COVID-19 survivors with 120 healthy participants without a history of COVID-19 showed that systolic blood pressure was significantly elevated in COVID-19 survivors [16]. It was observed in a longitudinal study of 354 recovered COVID-19 cases that, three months post-recovery, 5 individuals developed hypertension and that 51% of these individuals reported general symptoms of fatigue and persistent cough, which was statistically significant (*p* = 0.027) [11].

### 3.3. New-Onset Dyslipidaemia after COVID-19 Recovery

Table 3 summarises studies that investigated patients with persistent symptoms and altered lipid biochemistry for more than four weeks after recovering from initial SARS-CoV-2 infection; therefore, patients exhibited new-onset dyslipidaemia as part of the post-COVID condition. The prospective and observational cohort study by Dennis et al. showed significantly higher triglycerides, total cholesterol and low-density lipoprotein cholesterol (LDL-C) levels in individuals discharged four months prior from the hospital for COVID-19 compared with non-hospitalised individuals [17]. Similarly, six months after hospital discharge, a retrospective cohort study demonstrated that total cholesterol, LDL-C and high-density lipoprotein cholesterol (HDL-C) were significantly higher in those diagnosed with severe COVID-19 at admission [18]. A study by Gameil, M.A. et al. showed that amongst other biochemical alterations, triglycerides and LDL-C were significantly higher (*p* = 0.001) three months post-recovery from SARS-CoV-2 infection than in control peers with no history of COVID-19 [16].

## 4. Discussion

This systematic review compared studies that evaluated changes in glucose levels, blood pressure readings and lipid profiles after recovery from COVID-19 to verify the hypothesis that new-onset diabetes mellitus, arterial hypertension and dyslipidaemia are a possible sequela of a COVID-19 infection. Compiled studies investigating glucometabolic abnormalities showed that a select number of recovered COVID-19 patients were diagnosed with diabetes and had continuous hyperglycaemia several months after infection [9,10,11,12,13]. Similarly, a recent systematic meta-analysis that pooled analysis from four observational studies showed a 59% higher risk of developing incident diabetes in the post-COVID phase [19]. Selected studies evaluating new-onset hypertension in COVID-19 survivors demonstrated elevation in systolic blood pressure and minimal diastolic blood pressure changes one to three months after recovery [11,14,15,16]. Studies included in this review investigating lipid profiles revealed significant increases in triglycerides, LDL-C and total cholesterol levels in patients three to six months after being discharged from the hospital due to COVID-19 compared with those who did not require hospitalisation or had a milder initial infection [16,17,18]. Since the selected studies provided evidence of long-lasting physiological changes several months after COVID-19, it is paramount to further discuss the possible pathophysiology of SARS-CoV-2 infection for the effective clinical management of post-COVID patients.

Multiple hypotheses have been proposed to explain the association between new-onset diabetes and COVID-19 infection. A possible mechanism for altered glucometabolic control is SARS-CoV-2 damaging the pancreas. The angiotensin-converting enzyme 2 (ACE2) plays a crucial role in glucose homeostasis and insulin secretion by regulating beta-cell physiology. Within the pancreas, ACE2 is expressed within the pancreas’ endocrine islet cells, including beta cells [20,21]. ACE2 primary protein facilitating SARS-CoV-2 attachment and entry into the host cells [22,23]. Given the ability of SARS-CoV-2 to infect human-pluripotent-stem-cell-derived pancreatic cells in vitro and the presence of SARS-CoV-2 in pancreatic samples from COVID-19 patients, there is a strong suggestion that SARS-CoV-2 can invade the pancreas and directly cause pancreatic injury and diabetes by reprogramming cells to produce glucagon rather than insulin [23]. Secondly, SARS-CoV-2 triggers macrophage-mediated cytokine storm in which elevated levels of circulating cytokines and immune cell hyperactivation leads to excess inflammation facilitating insulin resistance and beta cell hyperstimulation. SARS-CoV-2 induces a decrease in the enzyme SETDB2 within macrophages, causing increased transcription of inflammatory cytokines leading to damage to the pancreas [24,25]. Autopsy tissue from patients who died of COVID-19 shown local inflammation caused by infections is associated with necroptotic cell death in islets causing islet damage [26]. Thirdly, alterations in post-translational protein modifications (PTMs) enables the breaking of central tolerance through the generation of neoepitopes that provide novel determinants that can activate T-cells that trigger autoimmune conditions like type 1 diabetes mellitus [27]. Alternatively, steroids are used as an anti-inflammatory treatment in patients with COVID-19. A recent Cochrane study showed that systemic corticosteroids probably reduce all-cause mortality in people hospitalised due to systematic COVID-19 [28]. Glucocorticoids worsen insulin resistance, sustain gluconeogenesis, worsen glycaemic control and cause marked hyperglycaemia. In turn, this hyperglycaemia leads to reduced insulin sensitivity and increasing insulin secretion, interfering with glucagon-like peptide 1 (GLP-1) and enhances the production of glucagon [29]. Unfortunately, the use of irrationally high doses of steroids in managing COVID-19 patients can lead to in-hospital glucocorticoid-induced hyperglycaemia (GIH). GIH is usually a temporary problem that resolves after discontinuing glucocorticoids, but data currently show that diabetes can persist and even unmask a pre-existing glucose metabolism disorder [30].

Hypertension is a major risk factor for stroke, coronary artery disease (CAD), renal disease, heart failure and peripheral vascular disease; therefore, early diagnosis and timely intervention are crucial in preventing these complications. Possible mechanisms explaining the development of new-onset hypertension due to SARS-CoV-2 infection include renin-angiotensin-aldosterone system (RAAS) dysregulation. It is hypothesised that SARS-CoV-2 binds to the ACE2 receptor via its spike (S) protein provoking transient ACE2 down-regulation consequently deregulating RAAS signalling [23]. ACE2 facilitates the cleaving of angiotensin II and counter-regulates the RAAS, resulting in a local accumulation of angiotensin II [31]. Angiotensin II leads to unfavourable effects, including vasoconstriction and hypertension, cellular differentiation and growth and inflammation [32]. Angiotensin II regulates NADPH oxidase activity, leading to the increased production of reactive oxygen species (ROS), further damaging the endothelium and ultimately leading to organ damage [31]. In addition, the imbalance leads to downregulation of the cardioprotective factors angiotensin 1-7 RAAS [33]. In summary, SARS-CoV-2 dysregulates RAAS raising angiotensin II levels and causing vasoconstriction, resulting in hypertension [33].

The measurement of plasma lipids and lipoproteins is critical in cardiovascular disease (CVD) risk management. Multiple possible mechanisms may explain why patients experience new-onset dyslipidaemia after COVID-19 infection. For one, SARS-CoV-2 is an enveloped virus meaning a lipid bilayer surrounds it; therefore, lipid metabolism plays a crucial role in the viral life cycle [33]. The virus utilises its lipid envelope for invasion and targets lipid synthesis and signal modification of host cells to generate lipids for its envelope [34]. Previous studies show that altered lipid metabolism occurs following infection with similarly structured viruses, indicating a biological relationship [30,35]. Secondly, lipids play a crucial role in modulating the immune system. SARS-CoV-2 induces a so-called “cytokine storm” due to the excessive activation of immune cells causing immune-mediated inflammatory dyslipoproteinaemia and causing immune cells to trigger a dysregulation of lipid production [35]. Low HDL levels prevent its ability to bind and neutralise pathogen-associated lipids that mediate the excessive immune activation leading to chronic inflammation [34]. Finally, the liver plays a vital role in lipid metabolism. The manifestation of liver injury caused by SARS-CoV-2 via liver ACE2/DPP-4 receptor typically involves decreased albumin and elevated aminotransferase and bilirubin [36,37].

Persistent pathophysiological and clinical alterations following COVID-19 infection call attention to the importance of mitigation strategies in COVID-19 prevention. A longitudinal observational study amongst vaccinated and unvaccinated SARS-CoV-2 infected healthcare workers not requiring hospitalisation in Italy showed lower prevalence of long-COVID amongst vaccinated [38]. Though association between vaccination against SARS-CoV-2 and long-COVID is important to consider in reducing the severity of COVID-19 and long-term effects.

## 5. Strengths and Limitations

This systematic review compared studies that evaluated changes in glucose levels, blood pressure readings and lipid profiles several weeks after recovery from COVID-19 to verify the hypothesis that new-onset diabetes mellitus, arterial hypertension and dyslipidaemia may be a sequela of a COVID-19 infection. The review had a couple of strengths, such as geographical diversity and the statistical validity of selected studies that compared clinical presentation at acute versus post-COVID period. The review also had some limitations, such as the selected studies did not distinguish between disease characterisation, such as diabetes type 1 or type 2. Another limitation of this review was the scarcity of studies investigating clinical presentation and biomarkers of post-COVID condition at the point in time the systemic review was conducted.

## 6. Conclusions

It can be concluded from this review that people older than eighteen years of age are at an increased risk of developing new-onset incidents of diabetes, hypertension, or dyslipidaemia several months after COVID-19 infection. In addition to other post-COVID symptoms, patients presented with altered glucose levels, blood pressure and lipid profiles several months after recovery from COVID-19 infection. Therefore, it is imperative that hyperglycaemia and insulin resistance, increased systolic blood pressure and altered lipid profiles be considered a part of the multifaceted post-COVID. Evidence-based clinical guidelines for the diagnosis and management of post-COVID syndrome that consider alteration in glucose levels, blood pressure and lipid profiles need to be established to provide patients with integrated clinical care, follow-up and monitoring after COVID-19 recovery. Further investigation of the multifactorial long term medical consequences after recovery from COVID-19 need to be conducted.

## Figures and Tables

**Table 1:** Summary of studies investigating new-onset diabetes mellitus after COVID-19 recovery.

Reference	Country	Study Type	Sample Size	Follow-Up Timeframe	Results
Montefusco, L. et al. [9]	Italy	Retrospective observational cohort study	551	Six months	Among 151 patients who exhibited new-onset hyperglycaemia at hospital admission for COVID-19, persistent hyperglycaemia continued to be observed in 52 (35%) patients, overt diabetes was diagnosed in about 2% of patients and the remaining 63% of patients showed remission and became normoglycemic.
Chen, M.et al. [10]	China	Prospective study	64	Three and six months	Fasting C-peptide [mmol/L] Mean ± SD Baseline 0.35 ± 0.24; 3-month follow-up 2.36 ± 0.98; 6-month follow-up 2.52 ± 1.11; *p*-value < 0.001. Fasting blood glucose [mmol/L] Mean ± SD Baseline 5.84 ± 1.21; 3-month follow-up 4.95 ± 0.76; 6-month follow-up 5.40 ± 0.68; *p*-value < 0.003.
Nesan, G. et al. [11]	India	Longitudinal study	354	Three months	Ten newly diagnosed with diabetes mellitus: six (66.6%) females and three (33.3%) males; *p* = 0.002.
Ayoubkhani, D. et al. [12]	England	Retrospective cohort study	47,780	Five months	Rate of new-onset diabetes was raised in post-COVID patients with 29 diagnoses per 1000 patient-years.
Huang, C. et al. [13]	China	Ambidirectional cohort study	1733	Six months	Fifty-eight patients without a self-reported history of diabetes were newly diagnosed with the condition at follow-up.

COVID-19, coronavirus disease 2019; mmol/L, millimoles per litre; *p*, *p*-value; SD, Standard Deviation.

**Table 2:** Summary of studies investigating new-onset hypertension after COVID-19 recovery.

Reference	Country	Study Type	Sample Size	Follow-Up Timeframe	Results
Akpek, M. [14]	Turkey	Retrospective cohort study	153	One-month	Systolic Blood Pressure [mmHg] mean ± (interquartile range) (*p* < 0.001) On admission 120.9 ± (113.7; 128.1); Post-COVID-19 126.5 ± (111.5; 141.5) Diastolic Blood Pressure, [mmHg] mean ± (interquartile range) (*p* < 0.001) On admission 78.5 ± (74.1; 82.9) Post-COVID-19 81.8 ± (74.4; 89.2)
De Lorenzo, R. et al. [15]	Italy	Retrospective and prospective cohort study	185	Twenty-three days	Systolic Blood Pressure [mm Hg] median (interquartile range) *p* = 0.17 All Cohort n = 185: 132.5 (123; 144.8); Discharged from ED *n* = 59: 130 (120;141); Hospitalized *n* =126: 134 (125;145); Diastolic Blood Pressure, [mmHg] median (interquartile range) *p* = 0.99 All Cohort *n* = 185: 70 (70; 85) Discharged from ED *n* = 59: 70 (70; 86); Hospitalized *n* =126: 80 (70; 85).
Gameil, M.A. et al. [16]	Egypt	Case-control study	120	Three months	Systolic Blood Pressure [mm Hg] mean ± SD *p* = 0.001 COVID-19 survivors 126.70 ± 10.31; Control group 120.63 ± 8.49. Diastolic Blood Pressure, [mmHg] mean ± SD *p* = 0.08 COVID-19 survivors 79.94 ± 7.32; Control group 77.86 ± 7.05.
Nesan, G. et al. [11]	India	Longitudinal study	354	Three months	5 individuals developed hypertension: 4 (80%) females and 1 (20%) male; *p* = 0.002.

COVID-19, coronavirus disease 2019; ED, emergency department; *n*, sample size; *p*, *p*-value; SD, standard deviation.

**Table 3:** Summary of studies investigating new-onset dyslipidaemia after COVID-19 recovery.

Reference	Country	Study Type	Sample Size	Follow-Up Timeframe	Results
Dennis A. et al. [17]	United Kingdom	Prospective, observational cohort study	201	Four months	30% (11 of 38) prior hospitalised individuals vs. 7.2% (12 of 163) non-hospitalised individuals reported high triglycerides (*p* = 0.002)**;** 60% (22 of 38) prior hospitalised individuals vs. 38% (61 of 163) non-hospitalised individuals reported high triglycerides (*p* = 0.04); 57% (19 of 38) prior hospitalised individuals vs. 31% (20 of 163) non-hospitalised individuals reported high LDL-C (*p* = 0.01)**.**
Li G. et al. [18]	China	Retrospective cohort study	107	Six months	Total cholesterol [mg/dL] Mean ± (interquartile range)Mild *p* = 0.042 ADM 197.9 (53); Follow-up 203.4 (33.6). Severe/Critical *p* = 0.016 ADM 192.4 (70.3); Follow-up 203.2 (94.5). Triglycerides [mg/dL] Mean ± (interquartile range)Mild *p* = 0.001 ADM 165.5 (88.5); Follow-up 140.8 (69). Severe/Critical *p* = 0.376 ADM 141.7 (74.6); Follow-up 143.9 (89). HDL-C [mg/dL] Mean ± (interquartile range)Mild *p*= 0.297 ADM 52.9 (18.2); Follow-up 54.5 (16.6). Severe/Critical *p* = 0.042 ADM 50.3 (21.7); Follow-up 55.3 (19.7). LDL-C [mg/dL] Mean ± (interquartile range)Mild *p* = 0.048 ADM 96.1 (32.9); Follow-up 103.6 (47.2). Severe/Critical *p* = 0.003 ADM 100.5 (26); Follow-up 103.6 (25.5).
Gameil, M.A. et al. [16]	Egypt	Case-control study	120	Three months	Total cholesterol [mg/dL] Mean ± SD *p* = 0.001 Cases 209.90 ± 32.04; Control 168.75 ± 33.22. Triglycerides [mg/dL] Mean ± SD *p* = 0.001 Cases 214.54 ± 62.21; Control 132.21 ± 43.48. HDL-C [mg/dL] Mean ± SD *p* = 0.119 Cases 42.98 ± 8.98; Control 45.78 ± 13.14. LDL-C [mg/dL] Mean ± SD *p* = 0.001 Cases 124.80 ± 30.06; Control 96.90 ± 30.83. VLDL-C [mg/dL] Mean ± SD *p* = 0.001 Cases 42.50 ± 12.21; Control 26.90 ± 10.69.

ADM, admission; COVID-19, coronavirus disease 2019; HDL-C, high-density lipoprotein cholesterol; LDL-C, low-density lipoprotein cholesterol; SARS-CoV-2, severe acute respiratory syndrome coronavirus 2; SD, standard deviation; VLDL-C, very low-density lipoprotein cholesterol.

## Data Availability

The data that support the findings of this review are available from the corresponding author upon reasonable request.

## Supplementary Materials

The following supporting information can be downloaded at: https://www.mdpi.com/article/10.3390/ijerph192013280/s1, Figure S1: PRISMA flowchart-Flow diagram of the review.

## Abbreviations

ADM	admission
ACE2	angiotensin-converting enzyme 2
BP	blood pressure
CVD	cardiovascular disease
COVID-19	coronavirus disease 2019
DBP	diastolic blood pressure
DPP-4	dipeptidyl peptidase-4 inhibitor
ED	emergency department
GIH	glucocorticoid-induced hyperglycaemia
GLP-1	glucagon-like peptide 1
HDL-C	high-density lipoprotein cholesterol
LDL-C	low-density lipoprotein cholesterol
Long-COVID	long-coronavirus disease
*n*	sample size
NADPH	reduced nicotinamide adenine dinucleotide phosphate
*p*	*p*-value
post-COVID	post-coronavirus disease
PTMs	post-translational protein modifications
RAAS	renin-angiotensin-aldosterone system
ROS	reactive oxygen species (ROS)
RT-PCR	real-time reverse-transcriptase polymerase chain reaction
SARS-CoV-2	severe acute respiratory syndrome coronavirus 2
SD	standard deviation
SETDB2	SET Domain Bifurcated Histone Lysine Methyltransferase 2
SBP	systolic blood pressure
VLDL-C	very low-density lipoprotein cholesterol
