# Supplementary figures and images for: New-Onset Diabetes Mellitus, Hypertension, Dyslipidaemia as Sequelae of COVID-19 Infection—Systematic Review
Source: Int J Environ Res Public Health. 2022 Oct 14;19(20):13280. doi: 10.3390/ijerph192013280 (PMC9602450; doi:10.3390/ijerph192013280)

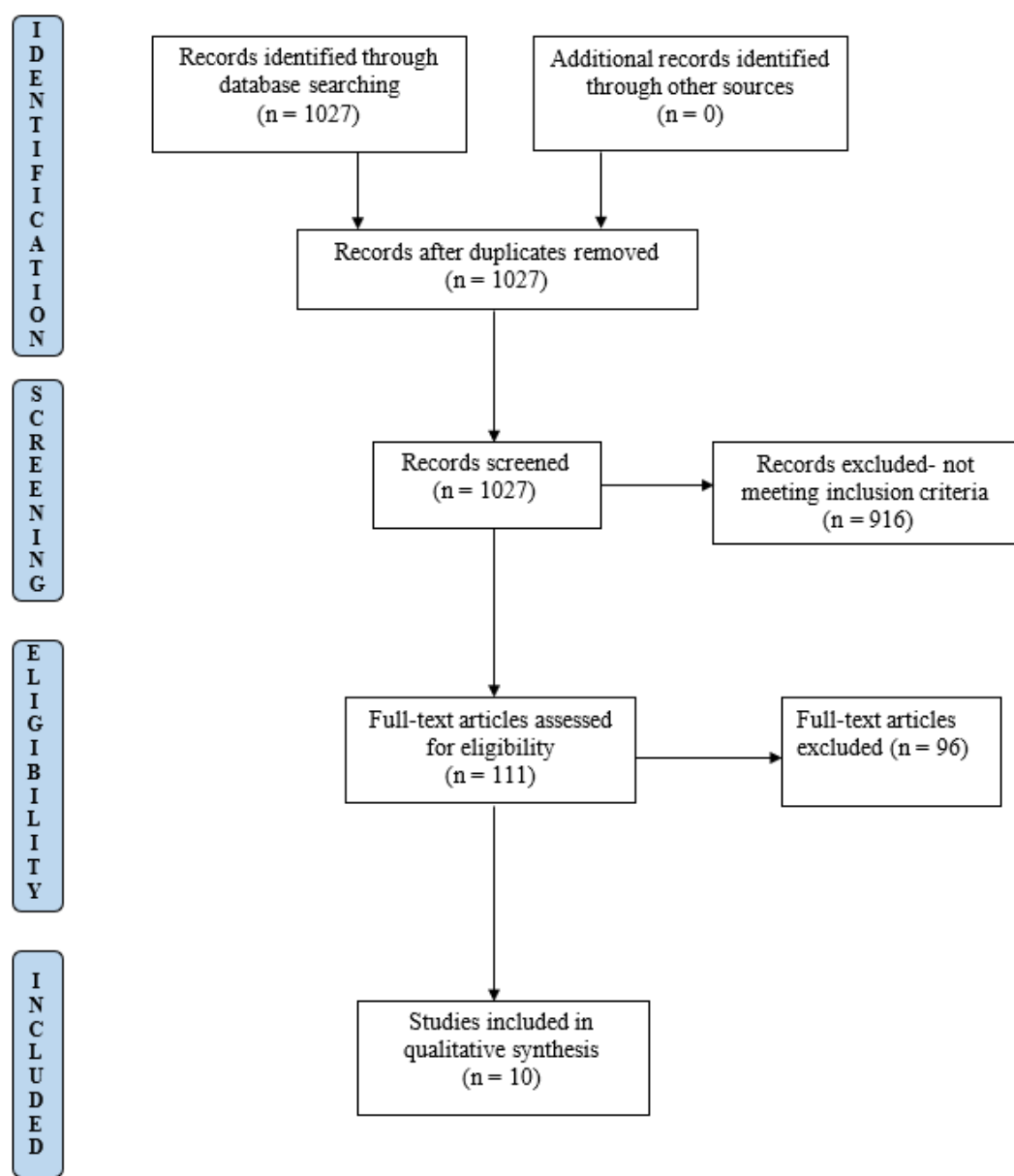

**Figure S1.** PRISMA flowchart- Flow diagram of the review.

Supplement: Supplementary file 1 [file ijerph-19-13280-s001.zip › ijerph-1879659-supplementary.pdf]
